# Safety and efficacy of airbag midwifery in promoting normal vaginal delivery and reducing caesarean section 

**Published:** 2012-11

**Authors:** Jianting Ma, Huajiang Shao, Xingren Lu, Bo Zhang, Guanger Zhang

**Affiliations:** *Department of Obstetrics and Gynecology, The People’s Hospital of Yuyao, Zhejiang 315400, China.*

**Keywords:** *Midwifery skill*, *Airbag*, *Efficacy*, *Safety*

## Abstract

**Background:** Balloon bionic midwifery has been applied in clinical obstetrics in China for 10 years, although played a certain role in controlling and improving the quality of obstetrics and caesarean section rate, but some questions have not been resolved.

**Objective: **The aim of this study was to investigate the efficacy and safety of airbag midwifery.

**Materials and Methods:** Primiparas (2410 cases) with various medical conditions were randomly divided into airbag and control groups undergoing the same obstetrical treatments, but airbag midwifery as a birthing option was chosen twice when the cervix was dilated to 2-4 cm during labor.

**Results:** The duration of the first and second stages, as well as the total delivery process, of the airbag group was shorter than that for the controls. The natural delivery rate of the airbag group was higher than that of the control group. The forcep delivery rate, cesarean section rate, amount of vaginal bleeding within 2 h after delivery, rate of postpartum hemorrhage, fetal distress, and pitocin use in the airbag group were all lower than those in the control group. No significant difference in the rate of maternal and fetal complications was observed in the two groups. The bionic airbag midwifery approach did not contribute to the incidence rate of urine retention, leukocyte count, neutrophil proportion, and level of creactive protein and IL-6 24 h after delivery.

**Conclusion:** Airbag midwifery skill is a simple, effective, and safe procedure.

## Introduction

Balloon midwifery, a novel midwifery technique, has been used in clinical obstetrics for more than 10 years in China (1-3). Although improvements in perinatal quality and control of cesarean section (CS) rates were shown by some studies, popularizing the technique remains difficult primarily because of the lack of large-scale specimens, multi-index data, and convincing scientific evidence. 

Thus, the advantages and clinical value of this childbirth technique have not been realized by most obstetricians. Therefore, we performed a clinical study on the application of bionic airbag midwifery in 2410 cases of natural vaginal delivery to verify its efficacy and safety in promoting natural vaginal labor and reducing CS rates. In recent years, the rate of CS delivery has constantly increased, specifically in China, because of iatrogenic and social factors; the CS delivery rate is even higher than that of natural delivery at some areas and hospitals (4-7). Although the increased CS rate has prompted positive action for the resolution of pregnancy complications (such as decreasing maternal and fetal morbidity and mortality rates in the perinatal stage), the cost of hospitalization and operative complications increase correspondingly. The incidence of anoxia in newborns has not been reduced and an increasing relative risk of maternal complications has also occurred (8-10). Therefore, the popularization and application of bionic airbag midwifery is one of the important and effective ways to improve the quality of natural vaginal delivery and control, and reduce the rate of CS delivery. On the basis of natural vaginal delivery and physical mechanisms, bionic airbag midwifery can stimulate the mechanical action of the fetal head and gradually dilate the vagina and cervix through inflatable balloon expansion. The puerperal status is transformed from primipara to “maternal” in a short time, thereby creating conditions favorable for a smooth delivery.

Artificial rupture of membranes followed by balloon dilatation causes the exposed parts of the fetus to drop and directly press on the lower segment of the uterine, cervix, and upper vagina, leading to more reflex contractions of the uterus. After the artificial rupture of the membranes, the concentrations of Ca^2+^ and prostaglandin in the serum and amniotic fluid increase, thereby promoting the influx of Ca^2+^. The increased Ca^2+^ of uterine smooth muscles cells may bind to actin and myosin to promote the contraction of the uterine smooth muscles. At the same time, the mechanical expansion of the vagina presses the rectal wall, causing feelings of defecation and forcing a fetal decline.

The duration of the first and second stages, as well as the total delivery process in the airbag group, was significantly shorter than that in controls. The rate of vaginal delivery in the observation group was higher than that in the controls, but the rates of forceps delivery and CS in the observation group were lower than those in the controls. Furthermore, the volume of postpartum blood loss (2h) and rates of postpartum hemorrhage, fetal distress, and induction using oxytocin injection in the observation group were statistically lower than those in the controls. The findings suggest that through the above-mentioned mechanisms, bionic airbag midwifery results in a more secure, convenient, better, and faster vaginal delivery. It also presents important clinical applications for improving the quality of vaginal delivery.

To ensure the safety and efficiency of bionic airbag midwifery, the following points should be considered: Indications and contraindications should be precisely controlled. Primiparas can be selected according to the following criteria: full-term pregnancy, cephalic presentation, a cervix Bishop score ≥8, no vaginal bleeding in late pregnancy, no reproductive tract infection during pregnancy, and no sexually transmitted diseases. Otherwise, it should be cautiously adopted or even forbidden as a form of treatment.

## Materials and methods

**Figure 1:**
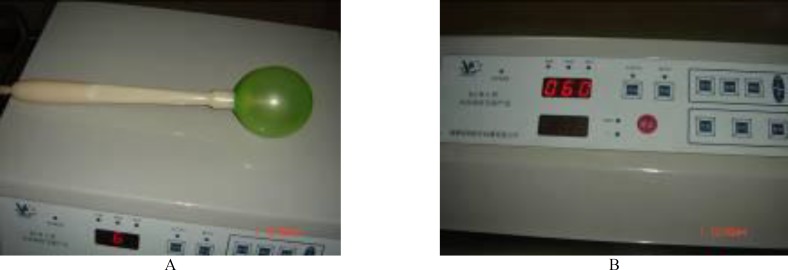
Illustration of the air bag bionic midwifery instrument. (A) Latex gasbag; (B) Control panel

The device, called a bionic airbag, is shown in Figure 1, which the price is about 20,000 Yuan RMB. In a randomized clinical trial, 2410 cases were chosen from primiparas admitted between May 2002 and September 2008 (Table I). The study was conducted in accordance with the Declaration of Helsinki and approval by the People’s Hospital of Yuyao Ethics Committee. Informed written consent was obtained from all subjects. 

According to the statistical data, the samples more than 1000 could satisfy the requirements. Most of the patients who underwent CS delivery were diagnosed with fetal distress and fetal position anomalies; a few were in labor for an extended period. The following criteria were applied at baseline: full-term singleton pregnancy, cephalic presentation, a cervix Bishop score≥8, and no gestational complications. 

**Figure 2:**
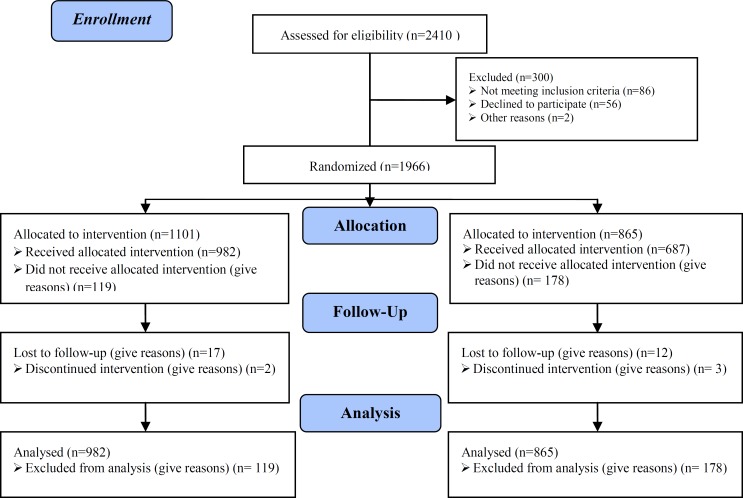
Consort flow diagram

Before delivery, the primiparas expressed their consent to receive the treatment. After primary screening, they were randomly assigned to two groups according to the random arrangement method; 1286 cases were classified as the observation group and 1124 were assigned in the control group. High-risk pregnancies were excluded from each group. The women in the experimental group underwent labor and airbag-assisted delivery, in which an Automatic Sac Practice Miduifeny Instrument NT-Q9501 (China). Briefly, when the uterine cervix dilated to 2-3 cm after regular uterine contractions, a sterile latex balloon was laid up to the superior endostoma of the cervix with a clamp along the cervical canal lateral wall. The balloon was operated according to the operating instructions: inflation rate was set to speed 2 (about 4 min), balloon diameter was set to 6-8 cm), cylinder pressure was at 0.3 MPa, balloon holding time was 5 min, and the cervical dilatation times considered was 1-2 (Figure 2). 

Every primipara needed to be observed by a specialist before and after treatment. The control group was given the same treatments as the observation group, except bionic airbag midwifery was not applied to the former. The clinical indications for CS were fetal distress and abnormal fetal position or extended labor. The indexes of evaluating obstetric quality during labor were delivery mode (natural delivery, forceps delivery, and CS), postpartum blood loss and rate of postpartum hemorrhage, rate of fetal distress, rate of neonatal asphyxia, rate of soft birth canal injury and episiotomy, routine blood examination 24 h after delivery, incidence of postpartum urinary retention, etc. 

**Statistical analysis**

The data were analyzed using SPSS (version 12.0, SPSS Inc., USA). Student’s T- test and Chi-square test were used for data analysis.

## Results

A total of 1286 patients in the observation group and 1124 in the controls with mean ages of 25.87±2.43 and 25.42±2.67 years, respectively, completed the study. Table I shows that the differences between the two groups were statistically non-significant in terms of duration of pregnancy. The results revealed that duration of the first and second stages, as well as the total delivery process, was shorter in the airbag group by 2.84, 0.29, and 2.92 h, respectively (Table II).

Our findings also showed that 76.32% of the observation group gave birth naturally, whereas only 61.12% of the controls underwent normal vaginal delivery. Conversely, the rates of forceps delivery and CS in the observation group were lower than those in the controls. The differences between the two groups were statistically significant (Table III). Vaginal bleeding was measured by volume methods 2 h after delivery, the difference was about 20 ml between two groups. The average volume of postpartum blood loss (2 h) and the rate of postpartum hemorrhage [242.97±69.37ml, 1.54% (17/1101)] in the observation group were significantly lower than those [262.50±82.01ml, 3.35% (29/865)] in the control group (p=0.034, <0.05). The U value was 2.21. The difference was statistically significant (p=0.0234, <0.05). Fifty-four cases in the observation group and 107 cases in the control group exhibited fetal distress; thirteen cases in the observation group and 17 cases in the control group presented with neonatal asphyxia. 

The rate of fetal distress in the observation group (4.20%, 54/1286) was significantly lower than that in the control group (9.52%, 107/1124) (p=0.0056, <0.01). However, no significant difference between the two groups (p=0.068, >0.05) was observed in terms of the rate of neonatal asphyxia. As shown in table IV, no statistically significant difference between the two groups (p>0.05) was found in terms of the rates of soft birth canal injury and episiotomy. Oxytocin injection was used for induction in the event of abnormal labor due to secondary uterine inertia. The rate of induction using oxytocin injection in the observation group (195/1286, 15.16%) was significantly lower than that in the controls (304/1124, 27.05%) (p=0.0048, <0.01). No statistically significant difference was found between the two groups (p=0.063, >0.05) in terms of the incidence of postpartum urinary retention (Table V).

No serious postpartum complications, such as postpartum puerperal infection, soft birth canal laceration, prolapsed umbilical cord, and fetal and infant mortality, occurred in the two groups.

**Table I:** Characteristics of the participants in experimental and control groups

	**Observation group** **(bionic air-bag midwifery)**	**Control group** **(conventional delivery)**
Number	1286	1124
Age (years)	25.87 ± 2.43	25.42 ± 2.67
Pregnant weeks	39.52 ± 1.44	39.24 ± 1.21
Times of pregnancy	1.62 ± 0.36	1.74 ± 0.66
Birth weight (g)	3338.27 ± 393.86	3322.16 ± 434.72

**Table II:** Different stages of labor in the observation and control groups

	**Observation group**		**Control group**	**p-value**
n*		1101	865	
The first stage (hour)		7.08 ± 2.24	9.92 ± 3.16	0.0053
The second stage (hour)	0.58 ± 0.27		0.87 ± 0.52	0.024
Total time (hour)	7.76 ± 3.16		10.68 ± 2.97	0.038

* note: 185 primiparas underwent routine cesarean section in the observation group and 259 in control group.

**Table III:** Comparison of delivery mode in the observation and control groups

	**Observation group** **n=1286(%)**	**Control group** **n=1124(%)**	**χ** ^2^ ** value**	**p-value**
Natural delivery	982 (76.36)	687 (61.12)	31.86	0.056
Forceps delivery	119 (9.25)	178 (15.84)	20.35	0.064
Cesarean section	185 (14.39)	259 (23.04)	5.18	0.048

**Table IV:** Comparison of the rates of soft birth canal injury and episiotomy in the two groups

	**Observation group** **n (%)**	**Control group** **n (%)**	**χ** ^2^ ** value**
Cases*	1101	865	
Perineal laceration with degree I	58 (5.27)	44 (5.09)	2.31
Perineal laceration with degree II	23 (2.09)	20 (2.32)	0.21
Cervical laceration	14 (1.27)	15 (1.73)	0.19
Soft birth canal injury	95 (8.63)	79 (9.13)	0.35
Episiotomy	730 (66.30)	593 (68.55)	0.28

* note: 185 primiparas underwent routine cesarean section in the observation group and 259 in control group.

**Table V:** The results of the incidence of postpartum urinary retention [n^1^/n^2^ (%)]*

	**n**	**Vatural delivery**	**Forceps delivery**	**Cesarean section**
Observation group	1286	26/982 (2.69)	6/119 (5.04)	9/185 (4.86)
Controls	1124	19/687 (2.77)	8/178 (4.50)	13/259 (5.02)

* n^1^: cases of delivery mode; n^2^: cases of postpartum urinary retention.

## Discussion

The safety that bionic airbag midwifery affords mother and fetus remains a debated issue. The main concerns are as follows: increased probability of soft birth canal injury and intrapartum/ postpartum hemorrhage because of the additional surgical operation required for balloon expansion in the soft birth canal within a short period; fetal distress and neonatal asphyxia caused by the mechanical stimulation to the exposed fetus; increased chances of infection because of operative trauma; easy occurrence of umbilical cord prolapse because of the cervical dilatation prior to the descent of the fetal head.

In this study, therefore, we performed a prospective, randomized, controlled trial to verify the safety of bionic airbag midwifery. The volume of postpartum blood loss (2h) and the rate of fetal distress in the observation group decreased significantly compared with the control group. 

No statistically significant differences were observed between the two groups in terms of the rates of soft birth canal injury, episiotomy, neonatal asphyxia, postpartum urinary retention, postpartum puerperal infection, umbilical cord prolapse, etc. In particular, no significant difference was found in two sensitive indices: the levels of serum CRP and IL-6, which represent the degree of inflammatory stress responses (11). This result is primarily attributed to the following factors: 

1) The timely artificial expansion of the cervix and vagina may increase the extension of the soft birth canal, reduce the dropping resistance of the exposed fetus, promote fetal descent, increase the intensity of reflex uterine contractions, leading to reduced labor duration and energy consumption. 

2) Bionic airbag midwifery may also shorten of the duration of placental blood flow caused by uterine contraction and soft birth canal extrusion to fetal head. 

3) The operation can reduce postpartum hemorrhage caused by uterine atony and fetal hypoxia. 

4) Under strictly sterile conditions, the flexible operation using a latex balloon in a short period (less than 45 min) does not excessively stimulate the fetus or lead to injury of the soft birth canal. Thus, it does not increase the possibility of trauma and infection of the mother and child. 

5) Given that delivery can be completed within a short period through the reduction in labor duration, airbag midwifery does not cause serious birth complications, such as umbilical cord prolapse. Bionic airbag midwifery was previously reported to shorten the duration of fetal head-induced pressure to the perineum, and reduce the rate of perineal damage (12).

The balloon must be placed correctly. When the cervix is dilated, the balloon should be positioned in the cervical canal and external orifice. When the lower segment of the vagina is expanded, the airbag should be placed at the upper vaginal opening. Basic safety and efficacy principles should be followed to avoid complications, such as laceration of the soft birth canal and umbilical cord prolapse. The speed of airbag inflation, diameter size, and holding time must be considered according to the surgeon’s experience and the patient’s uterine contractions. Strict attention should be paid to aseptic manipulation during operation. 

The conditions of the patient, such as uterine contractions and variations in fetal heartbeat, must be closely observed. To ensure the smooth implementation of bionic airbag midwifery, a primipara can be given 5 ml of 2% lidocaine and 0.5 mg of atropine to block the cervix; the patient can also be given 10 mg of diazepam, which is injected into the cervix before the delivery is performed (13). 

This approach can enhance the effect of balloon dilatation, but also reduce discomfort from the partial bulge. For patients with maternal psychological stress, entonox inhalational analgesia can be used during labor to reduce pain during operation (15, 16). In conclusion, bionic airbag midwifery visibly reduces the duration of delivery, amount of bleeding, and pain during operation, among other benefits. It can also promote the rate of natural vaginal labor and imposes no negative effects on both mother and neonate. Although aspects related to long-term safety, such as the functional effect on the pelvic floor requires further investigation, bionic airbag midwifery is a safe and effective method recommended for use in clinical practice. 

## Conflict of interest

The authors have no conflict of interest regarding this paper.

## Footnote

***Registration ID in UMIN: UMIN0000060962011/09/01 ***
